# The impact of ICT-enabled extension campaign on farmers’ knowledge and management of fall armyworm in Uganda

**DOI:** 10.1371/journal.pone.0220844

**Published:** 2019-08-21

**Authors:** Justice A. Tambo, Caroline Aliamo, Tamsin Davis, Idah Mugambi, Dannie Romney, David O. Onyango, Monica Kansiime, Christine Alokit, Stephen T. Byantwale

**Affiliations:** 1 Centre for Agriculture and Biosciences International (CABI), Delémont, Switzerland; 2 Centre for Agriculture and Biosciences International (CABI), Entebbe, Uganda; 3 Centre for Agriculture and Biosciences International (CABI), Wallingford, England, United Kingdom; 4 Centre for Agriculture and Biosciences International (CABI), Nairobi, Kenya; 5 Ministry of Agriculture, Animal Industry and Fisheries (MAAIF), Entebbe, Uganda; CMAVE, USDA-ARS, UNITED STATES

## Abstract

This study evaluates the unique and combined effects of three complementary ICT-based extension methods ― interactive radio, mobile SMS messages and village-based video screenings ― on farmers’ knowledge and management of fall armyworm (FAW), an invasive pest of maize that is threatening food security in sub-Saharan Africa and Asia. Building on a survey of maize farmers in western Uganda and using various selection-on-observables estimators, we find consistent evidence that participation in the ICT-based extension campaigns significantly increases farmers’ knowledge about FAW and stimulates the adoption of agricultural technologies and practices for the management of the pest. We also show that exposure to multiple campaign channels yields significantly higher outcomes than exposure to a single channel, with some evidence of additive effects. These results are robust to alternative estimators and also to hidden bias. Results further suggest that among the three ICT channels, radio has greater reach, video exerts a stronger impact on the outcome measures, and greater gains are achieved when video is complemented by radio. Our findings imply that complementary ICT-based extension campaigns (particularly those that allow both verbal and visual communication) hold great potential to improve farmers’ knowledge and trigger behavioural changes in the identification, monitoring and sustainable management of a new invasive pest, such as FAW.

## Introduction

Fall armyworm (*Spodoptera frugiperda)* is a devastating crop pest native to tropical and sub-tropical regions of the Americas. It was first detected in West Africa in early 2016 [1], and given its capacity to migrate long distances and the suitable agro-climatic conditions in tropical Africa, the presence of the pest has been confirmed in almost all countries in sub-Saharan Africa (SSA) and is now spreading rapidly in Asia [2, 3]. FAW can feed on over 350 plant species [4], with maize being one of the most preferred hosts [5]. A study by Day et al. [6] has shown that in the absence of effective control methods, the pest has the potential to cause annual maize losses of about 8 to 20 million tonnes in just 12 of the maize-producing countries in SSA. Considering that many smallholders in SSA depend on maize production for food and subsistence [7], the pest poses a serious threat to food security and livelihoods in the region.

Typical of a new alien invasive pest, limited awareness and knowledge of FAW among farmers and key stakeholders can hamper successful management of the pest. For instance, without sufficient knowledge on FAW identification, the pest may be misconstrued for other common species of armyworms or caterpillar pests in Africa such as the African armyworm, maize stalk borer and African bollworm, potentially resulting in the application of ineffective management practices and a rapid build-up of the pest population [8]. Consequently, one of the immediate recommendations for mitigating the devastating effects of FAW is the need for rapid awareness raising programmes on how to identify, prevent and control the pest [6, 8].

Extension workers are important providers of agricultural information and advisory services to farmers in SSA [9], and their roles are particularly crucial when farmers face new challenges, such as the outbreak of FAW. However, they may be limited in their capacity to provide timely and actionable information to a large number of farmers due to challenges such as high farmer to extension worker ratio, poor infrastructure (and thus difficulty to reach remote areas), and low motivation and accountability [10–12]. For instance, only 19% of farm households in Uganda have contacts with extension workers [13]. The upsurge in information and communication technologies (ICTs) in the developing world in recent decades offers the opportunity to overcome many of these shortcomings and to complement traditional extension systems [11; 14]. Unlike conventional face-to-face extension approaches such as training and visit (T&V) and farmer field schools (FFS), ICT-based extension advisory methods enables broader and timely outreach to farmers, often in a cost-effective and interactive manner [8, 15]

In this paper, we assess the impact of an ICT-enabled extension campaign that was used to promote the awareness, identification, prevention and control of FAW to farmers and stakeholders in western Uganda, where maize is an important staple crop and FAW is causing deleterious impact. “An extension campaign is a coordinated effort to inform many farmers in a relatively short period of time about an agricultural topic of widespread concern or interest” [16]. The campaign used three different ICT applications: interactive radio, mobile phone short message service (SMS) and video screening. It was hoped that the farmers’ knowledge of FAW and propensity to adopt management practices will be enhanced significantly by exploiting the complementarities between the three ICT channels. For instance, both radio and video are suitable for awareness creation and disseminating information, but radio reaches a larger audience, while video allows visual communication and enhances learning [15, 17]. On the other hand, SMS messages are more appropriate for literate farmers and can be kept as a reference source and accessed at anywhere and anytime.

The improved access to radio, video and mobile phone-based services in rural areas have sparked interest in studying their potential and effectiveness in improving agricultural outcomes. For example, Hudson et al. [18] have shown that participatory radio campaigns implemented by Farm Radio International (FRI) have resulted in increased knowledge and adoption of promoted agricultural practices in six African countries, including Uganda. Similarly, studies have demonstrated that video-mediated extension generate positive outcomes, such as increased farmers’ knowledge and adoption of improved or sustainable agricultural practices and technologies [19–22], and strengthened innovation-generating capacity [17, 23]. A review of the literature on the impact of mobile phone based-services on farmers in developing countries by Baumüller [24] revealed limited and inconclusive empirical evidence. For instance, Fafchamps and Minten [25] found that receipt of SMS messages did not have significant impacts on Indian farmers’ likelihood of changing crop varieties and cultivation practices. By contrast, studies have shown that mobile phone-based services are associated with improved farmers’ knowledge and adoption of new agricultural practices [26, 27]; production of more diversified crops [28]; increased prices received by farmers [29]; and gender equality and improved household welfare [30].

Our study contributes to the literature on the effectiveness of ICT-based agricultural extension by focussing on farmers’ knowledge and management of a new invasive pest (FAW) that is wreaking havoc across SSA. Furthermore, we add to the literature by comparing three ICTs― mobile phone, radio and video. Most previous studies on the impact of ICT-mediated interventions have analysed these three ICT tools in isolation. In contrast, our study is based on an intervention that allows us to explore their unique and combined effects. The research questions addressed in this study include: (1) what factors influence participation in the ICT-mediated extension campaign on FAW?; (2) does participation in the campaign enhance farmers’ knowledge on how to identify, monitor and manage FAW?; (3) what is the impact of the campaign on the adoption of agricultural technologies and practices for the management of FAW?; (4) does exposure to multiple ICT-enabled campaign channels result in larger gains (in terms of knowledge and management of FAW) than exposure to only one channel?; and (5) to what extent are there differential effects of the three ICT-enabled extension approaches used in the FAW campaign?

## Materials and methods

### The FAW extension campaign

The FAW pest was first detected in Uganda in June 2016, and by the end of 2017, it had spread to all the districts of the country [31]. As part of efforts to combat the pest, an ICT-based extension campaign was implemented between March and August 2018 with the aim of creating awareness on the identification, prevention and management of FAW in Buliisa, Masindi and Kiryandongo districts in mid-western Uganda, where maize is widely cultivated. The campaign was closely aligned with the seasonal calendar of maize production in the region and was implemented through close collaboration between the campaign partners ꟷ Uganda’s Ministry of Agriculture, Animal Industries and Fisheries (MAAIF), District Local Governments, National Agriculture Research Organisation (NARO), CABI, Farm Radio International (FRI), Peripheral Vision International (PVI), and Hamwe East Africa Limited.

A technical brief on FAW monitoring, identification and management options was developed by the campaign team and subsequently validated by the Uganda FAW National Task Force, forming the basis for the messaging communicated through the campaign. The technical brief reflected nationally agreed recommendations as well as farmers’ information needs on the FAW pest. Prior to the launch of the campaign, a stakeholder consultation meeting was held among the implementing partners to design the campaign messages. The communication channels used for the campaign include interactive radio, video screenings and mobile SMS messaging, which were led by FRI, PVI and Hamwe, respectively. The three partners worked closely together to complement and promote each other’s’ activities in the campaign.

FRI developed a 10-week interactive series that ran on Radio Kitara, and was aired in Runyoro, the dominant language in mid-western Uganda. The campaign consisted of a one-hour live interactive radio programme and a repeat broadcast per week for 10 weeks; and around eight short spot messages per week. Each broadcast featured recorded information about an aspect of FAW, live interviews with two experts, as well as with farmers’ who shared their experiences about the pest. This was followed by phone-ins during which the experts responded to farmers’ queries. The radio series also publicised the video screening locations and schedules and advertised the SMS short code for participating in the SMS campaign. The radio campaign is estimated to have reached nearly 330,000 farmers [32].

PVI produced and disseminated an eight minute video (in both English and Runyoro) on FAW through community–based screenings in the three campaign districts using two media delivery tools: *Kibanda Boda and CrowdPullerz*. *Kibanda Boda* is a solar powered micro cinema that is mounted to the back of a motorcycle, and is able to reach farmers in remote communities. A typical *Kibanda Boda* video screening in a community was attended by 25–32 farmers, who were facilitated by a FAW content expert. Screening sessions lasted between 60 to 90 minutes, allowing ample time for question and answer sessions to ensure full comprehension of the topics among all participants. *CrowdPullerz*, on other hand, is an edutainment DVD-video that featured latest popular local music videos interspaced with a four-part video on FAW prevention, monitoring, and management as well as safe use of chemicals. The DVDs were distributed freely to 1,043 public screens (bars, salons, retail shops, buses, cinema halls, etc.) in the three campaign districts. Attendance lists from the *Kibanda Boda* screenings indicate that 4287 people participated in the video screenings, while the *CrowdPullerz* distribution lists suggest that about 36344 people watched the video through this platform across the three districts.

A total of 45 structured SMS messages in Runyoro language were pushed to a pre-existing database of 30,000 farmers by Hamwe. The database contained mobile phone numbers of some farmers in the study region who had already subscribed to receive maize-related contents from Hamwe. Additionally, a call-to-action approach was used where farmers were informed through the radio and video campaigns to send the word ‘FAW’ to a specified short code to receive FAW-related information, and about 2500 farmers did so. The farmers received three to five text messages per week consistently over a 10-week period.

### Household survey

Data for this study were collected between October and November 2018 through a survey of 607 maize producing households in mid-western Uganda, where the FAW campaign was implemented. The campaign was concentrated in three districts, namely Kiryandongo, Masindi and Buliisa. A multi-stage sampling approach, involving purposive sampling of districts, sub-counties, and random sampling of communities and farm households was adopted in this study. First, Masindi and Buliisa were selected from the three campaign districts because the inhabitants speak Runyoro, which was the primary language for the campaign. Kiryandongo district was excluded, as language barrier could have affected the quality of the campaign in this district, given that five different languages are spoken in the district. Within each district, we purposively selected three sub-counties based on the importance of maize production and the geographic coverage of Radio Kitara, which aired the radio campaign messages. The selected sub-counties include Bwijanga, Mirya and Pakanyi in Masindi district, and Biiso, Kihungya and Ngwedo in Buliisa district. Within each sub-county, we randomly selected between four to eight communities based on the size of the sub-county. Our data show that there were video screenings in at least two of the selected communities per sub-county. Finally, we randomly selected and interviewed about 10 to 20 farm households per community depending on the size of the community. In total, our sample consists of 202 and 405 households from Buliisa and Masindi districts, respectively. The sample households cultivate maize and had experienced FAW attack on their maize plots prior to the campaign.

The data were collected by 16 trained enumerators using a structured questionnaire that was pre-tested in Kigumba sub-county in Kiryandongo district. The questionnaire (S1 File) and data (S2 File) are available online. Before starting the interviews, the enumerators explained the purpose of the survey to the respondents and stressed that participation was voluntary. The respondents were asked to decide whether or not to participate in the survey, and this information was recorded on the questionnaire by the enumerators. Consent of each respondent was obtained orally, given that many of the surveyed farmers were illiterate. The questionnaire contained modules on participation in the FAW campaign; household socio-economic characteristics; maize production details; knowledge of and attitude towards FAW; adoption of interventions for FAW management; access and proximity to institutional support services; and the rational use of pesticides. The questionnaires were coded on the Open Data Kit (ODK) platform, utilising inbuilt data validation features that ensured data quality was not compromised. Interviews were conducted face-to-face using tablets. The data were received on an aggregate server in near real-time, and this made it possible to perform quality checks and provide feedback to enumerators, further ensuring data quality.

### Empirical approach

As is typical of impact assessments, the challenge of analysing the impact of the FAW campaign is determining what would have happened in the absence of the campaign. For any differences in the outcomes of interest to be attributed to the FAW campaign, the control group (non-participants) would need to be similar to the treatment group (campaign participants), except they would have not received the FAW campaign messages. Thus, both the treatment and control groups should have a similar range of observable characteristics (e.g., gender, education and wealth) and unobservable characteristics, such as entrepreneurial ability and motivation. The best way to obtain these comparable groups is to use an experimental design in which prior to the beginning of the extension campaign, farmers are randomly assigned to treatments groups (those who will receive information on FAW through one of the communication channels) and a control group (those who will not participate in the campaign). The experimental approach was, however, not possible in our case as farmers were not randomly assigned to participate in the extension campaign. Instead, our study is based on observational data where households self-selected into the extension campaign. We use propensity score matching (PSM) and doubly robust estimators to mitigate selection bias when evaluating the impact of the FAW extension campaign.

In this study, we are interested in assessing if FAW extension campaign increased farmers’ knowledge of FAW and their likelihood of adopting management practices. To address this objective, we first pool all the households that were exposed to the campaign regardless of the channels into one treatment group and compared them with households that were not exposed to any of the campaign channels (control group). We then separated the treatment group into subgroups based on the campaign channel(s) which the households were exposed to, and compared each of these subgroups to the control group. Thus, the second part of our analysis comprises multiple treatment assignments, as households who participated in the extension campaign had the possibility of obtaining the campaign messages through the following channels: (1) radio only; (2) SMS only; (3) video only; (4) radio and SMS only (Radio+SMS); (5) radio and video only (Radio+Video); (6) SMS and video only (SMS+Video); and (7) the three platforms of radio, SMS and video (Radio+SMS+Video). Our estimation techniques—PSM and doubly robust estimator― can handle both binary and multi-valued treatments.

PSM involves identifying non-participants of the campaign who are similar to participants in their observable characteristics. In the first stage of the PSM procedure, we generate propensity scores from a logit regression, which indicate the probability of a farm household participating in the FAW campaign. In the PSM with multi-valued treatments approach, we follow Lechner [33] by estimating separate conditional probabilities between participants of a single or a combination of campaign platforms and non-participants to obtain propensity scores using logit regressions. The covariates in the logit regression consist of important pre-treatment variables that could influence participating in the campaigns and the outcome variables. This includes household socio-demographic characteristics such as age, gender and education of household head, household size, dependency ratio, risk attitude, access to off-farm job and poverty level; information-related variables such as ownership of radio and mobile phones, access to extension services and membership in farmer group; and location characteristics, such as proximity to input market and district dummy. A detailed description of the control variables are presented in Table 1. We then use the propensity scores obtained in the first stage to match the campaign participants and non-participants. The matching method used is kernel matching, with the default bandwidth of 0.06. Kernel matching uses a weighted average of the non-participants to construct the counterfactual (unobserved) outcome, and the weight depends on the distance on the propensity score between the participants and non-participants [34]. To check the robustness of our results, we also used two alternative matching techniques, which we present later. After confirming that the distribution of covariates between participants of the various campaigns and non-participants are balanced, we estimate the treatment effects of the FAW campaign in the region of common support, where the propensity score distributions of treatment and control households overlap. The reliability of our PSM results hinges on the selection on observables or conditional independent assumption (CIA), which suggests that conditional on observable variables, potential outcomes are independent of treatment assignment [35]. We use the bounding approach proposed by Rosenbaum [36] to examine the robustness of our results against the violation of this assumption.

**Table 1:** Definition and summary statistics of covariates.

Variable	Description	Full sample		Participants		Non-participants	
		Mean	SD	Mean	SD	Mean	SD
Age	Age of household head (years)	43.25	13.63	43.18	13.81	43.46	13.08
Gender	Gender of household head (1 = male)	0.85	0.36	0.86**	0.34	0.79	0.41
Education	Number of years of formal education of household head	7.34	3.76	7.57***	3.53	6.62	4.34
Household size	Number of household members	6.82	3.30	6.77	3.30	6.99	3.91
Dependency ratio	Household dependency ratio^a^	1.39	1.17	1.33**	1.12	1.58	1.28
Land holding	Amount of land owned by household (hectares)	3.54	7.45	3.82	7.54	2.66	7.10
Input market	Distance from household to the nearest input shop (km)	3.93	5.35	4.09	5.70	3.40	4.08
Radio	Household owns radio (1 = yes)	0.85	0.36	0.91***	0.28	0.66	0.48
Phone	Household owns mobile phone (1 = yes)	0.89	0.32	0.92***	0.27	0.79	0.41
Extension access	Household has contact with extension agents (1 = yes)	0.28	0.45	0.32***	0.47	0.16	0.37
Farmer group	A household member belongs to a farmer association (1 = yes)	0.28	0.45	0.32***	0.47	0.16	0.36
Off-farm activity	Household member has an off-farm job (1 = yes)	0.49	0.50	0.51**	0.50	0.41	0.49
PPI	Poverty Probability Index (0–100)^b^	47.42	12.55	48.64***	11.82	43.58	13.97
Risk preference	Risk attitude of household following Dohmen et al. [44] (0–10)^c^	5.11	2.84	5.29***	2.90	4.55	2.59
District	Location of household (1 = Masindi; 0 = Buliisa)	0.67	0.47	0.71***	0.45	0.54	0.50
	Number of observations	607		460		147	

Notes

*** and ** indicate that the mean values for campaign participants are significantly different from non-participants at the 1%, 5% and 10% significance levels, respectively.

^a^A household’s dependency ratio is computed by dividing the number of household members under 15 years of age plus the number of members over 64 years of age by the total number of household members.

^b^The PPI is a simple country-specific asset-based poverty assessment [43]. The index is computed using a scorecard containing 10 indicators related to household characteristics, dwelling characteristics and ownership of durable assets, and it estimates the probability that a household consumption is below a given poverty line [42]. The PPI score ranges from 0 (household is most likely to be below a poverty line) to 100 (household is least likely to be below a poverty line). We used the PPI scorecard for Uganda.

^c^ 0 means not at all willing to take risks and 10 means fully prepared to take risks.

Our second estimation technique, the doubly robust estimator, models the treatment and outcome simultaneously to attenuate selection bias from non-random treatment assignment [37]. Among the class of doubly robust estimators, we employ the inverse-probability-weighted regression-adjustment (IPWRA) approach, which uses weighted regression coefficients to compute the treatment effect, where the weights are the estimated inverse probabilities of treatment [38]. Using the IPWRA approach to estimate the treatment effects of the FAW campaign follows three steps [39]. First, the probability of participating in the FAW campaign (i.e., the treatment model) is estimated using logit model (or multinomial logit regression in the case of multi-valued treatment assignments), and the predicted probabilities are used in computing the inverse-probability weights. Second, using these inverse-probability weights, weighted regression models of the outcome are fitted to obtain the expected outcomes of the probabilities of participating or not participating in the campaign. Finally, the mean outcomes for participating and non-participating households are computed, and the difference between these two means provides the estimates of the treatment effects of participating in the campaign. A key attractive feature of the IPWRA approach is its double-robust property, which allows the treatment effect to be consistently estimated as long as either the treatment model or the outcome model is correctly specified [37, 38]. In other words, this method is robust to misspecification in either the treatment model or the outcome model.

In both the PSM and doubly robust estimators, we are interested in estimating the average treatment effect on the treated (ATT), which measures how the FAW extension campaign affects the outcomes for households who decided to participate in the campaign. This can be expressed as:

ATT=E{Y(1)−Y(0)|C=1}=E{Y(1)|C=1}−E{Y(0)|C=1} (1)

where *Y*(1) and *Y*(0) are the outcomes for the campaign participants and non-participants, respectively; *C* denotes participating in the FAW campaign. Note that in the case of multi-valued treatments, *C* indicates participation in a particular campaign channel.

### Outcome variables

The impact of the extension campaign was assessed on outcome indicators related to farmers’ knowledge of FAW and the subsequent adoption of management practices. To assess FAW knowledge, the sample farmers were asked to respond to 30 questions related to FAW awareness, identification, monitoring and management. We generate four different knowledge scores from the 30 FAW knowledge questions. The first score includes a subset of 10 questions that relate to common myths about FAW, key morphological features of FAW and visible signs of damage caused by the pest. We refer to this score simply as the FAW identification score. The second score, which we term FAW monitoring score, includes a subset of five questions on when and how to monitor maize farms for the presence of FAW. A subset of 15 questions related to the integrated management of FAW forms the third score, and is referred to as the FAW management score. Ten of the 15 questions in this subset are related to the safe use of pesticides, given that pesticides are the most commonly applied FAW management option by African farmers [3, 40], but are toxic to humans and the environment. The final score, the overall FAW knowledge score, is the sum of the above three subscores, and thus comprises all the 30 knowledge questions. The number of correct answers to the questions within each score category represents the knowledge scores for that category. A summary of the 30 FAW knowledge questions is displayed in Table 2. The detailed questions are available upon request. It should be noted that some of the knowledge questions in the survey were negated to avoid monotonous responses.

**Table 2:** Summary statistics for FAW knowledge questions and scores (% correct responses).

Description	Total sample	Participants	Non-participants
	(n = 607)	(n = 460)	(n = 147)
***Questions on FAW awareness and identification***			
1. FAW attacks only maize	47.78	51.09***	37.41
2. FAW comes from or is spread through seeds	46.29	51.09***	31.29
3. FAW can cause 100% loss of maize yield	87.64	90.00***	80.27
4. FAW attacks all stages of maize growth	74.79	75.65	72.11
5. Colour of FAW egg masses	78.42	81.96***	67.35
6. Colour of FAW caterpillar	71.99	76.09***	59.18
7. Y-shaped mark on forehead of the caterpillar	59.97	63.91***	47.62
8. Four dark spots on tail end of the caterpillar	70.84	76.09***	54.42
9. Signs of FAW damage on maize leaves	98.85	99.57***	96.60
10. Presence of frass on heavily infested plants	91.27	95.22***	78.91
***Questions on FAW monitoring***			
1. Early detection allows early control and less damage	94.40	95.65**	90.48
2. Check for the presence of FAW 2–3 weeks after planting	83.86	86.52***	75.51
3. Continuously monitor farm every 3 days for FAW signs	85.00	89.57***	70.75
4. Walk along the edges of farm to monitor FAW	73.64	74.78	70.07
5. Number of plants to monitor per area	70.35	74.78***	56.46
***Questions on FAW management***			
1. Handpicking can be an effective control measure	59.47	63.48***	46.94
2. Number of infested plants before taking action	74.79	79.13***	61.22
3. Early planting can prevent or reduce FAW infestation	73.81	78.70***	58.50
4. Crop rotation and intercropping can reduce infestation	46.29	50.87***	31.97
5. Regular weeding can help prevent FAW infestation	35.26	40.00***	20.41
6. Chemical pesticides are not dangerous to humans	74.96	77.39**	67.35
7. Important to use PPE when mixing /spraying pesticides	93.57	95.43***	87.76
8. Spray pesticides when there are signs of rain	79.24	81.74***	71.43
9. Most effective time to spray pesticide to control FAW	83.20	85.22**	76.87
10. Reuse empty pesticide containers for other purposes	83.20	84.78*	78.23
11. Control FAW by spraying into the maize funnel only	42.34	43.26	39.46
12. Do not spray pesticides when maize is mature	73.97	73.91	74.15
13. Mix different pesticides to make them more effective	43.16	45.43**	36.05
14. Dosage of pesticides to apply	73.97	73.91	74.15
15. Monitor and re-spray affected plants within 7–14 days	82.21	85.87***	70.75
***Knowledge scores***			
Knowledge related to FAW awareness and identification	72.80	76.07***	62.52
Knowledge related to FAW monitoring	81.40	84.20***	72.60
Knowledge related to FAW management	67.87	70.67***	59.13
Overall FAW knowledge score	71.40	74.73***	62.53

Note

***, **, * indicate significant differences in the average score between campaign participants and non-participants at the 1%, 5% and 10% significance levels, respectively

One of the key objectives of the extension campaign was to foster the uptake of appropriate FAW management practices among farmers in the study region. To this end, the campaign participants were advised to use a combination of prevention and control practices to effectively manage the FAW pest. Following the principles of integrated pest management (IPM) [41], the recommended FAW management practices included cultural practices, biological control methods, local innovations, as well as rational use of pesticides. Our main indicator for the adoption of FAW management practices is, therefore, measured as the number of prevention and control practices that a household has adopted for the management of FAW on maize. We also analyse the adoption of some specific management practices.

## Results and discussion

### Descriptive statistics

**Fig 1:**
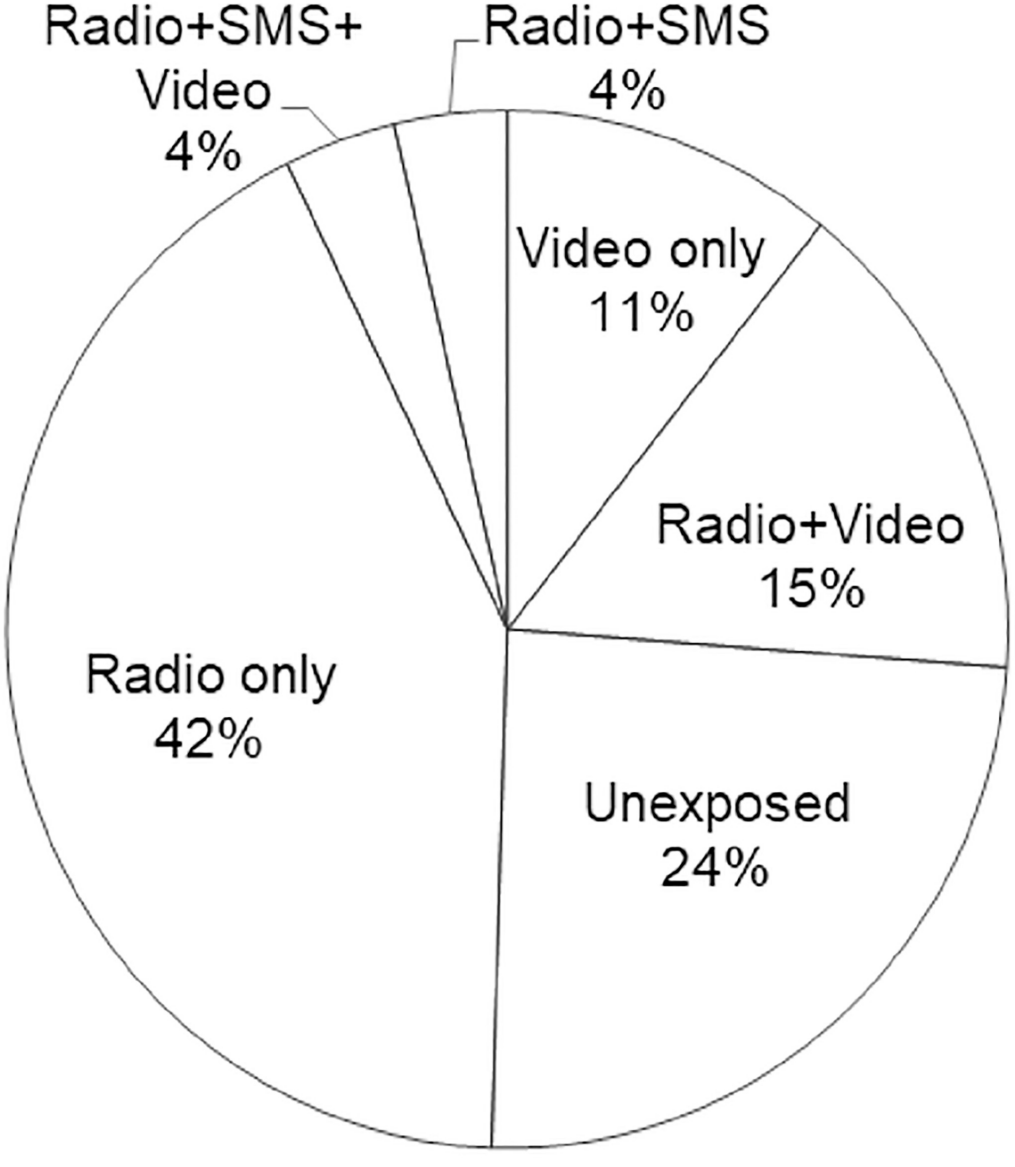
Percentage of households exposed to the major campaign channels (n = 603). Fig note: Two households each were exposed to SMS only and SMS+Video, making a total of 607 sample households.

Fig 1 depicts households’ exposure to the different campaign channels, which constitute our multi-valued treatment groups. Fifty-three percent were exposed to only one channel, 23% were exposed to at least two channels, while 24% were not reached by the campaign. Radio had the highest coverage, with nearly two-thirds of the households participating in the radio campaign. This is expected as radio is considered to be the most widely used source of information in rural Africa [18]. The video campaign reached 30% of our sample population, whereas exposure to SMS was low (8%). In terms of exposure to multiple campaigns, the combination of radio and video achieved the highest reach of 15%.

Table 1 reports descriptive statistics for the sample households. The average household head is 43 years old and has completed only seven years of formal education. On average, households consist of seven members, and 15% of the households are female-headed. The average land holding per household is about 3.5 hectares. Majority of the households own radio and mobile phones, which are important sources of agricultural information. By contrast, only 28% of the households were visited by an extension worker in the past 12 months prior to the campaign. This is consistent with the share of agricultural households in western Uganda who have contacts with extension workers, which is estimated to be 29% [13]. This strongly reinforces the potential importance of ICT-enabled extension services in complementing conventional extension services so as to expand the reach of agricultural information. Nearly half of the households in our sample engage in off-farm income generating activities. The households’ PPI scores, when converted to poverty likelihoods using look-up tables that come along with the PPI scorecard [42], suggest that our sample households have on average 19% likelihood of living below the international poverty line of $1.90 per day.

Table 1 further presents the results of tests of mean differences between campaign participants and non-participants. There are some significant differences between the two groups. For instance, compared to non-participating households, campaign participants have better educated household heads, have lower dependency ratios, are less risk averse and have lower poverty incidence. Further, campaign participants have better access to communication channels and sources of information such as radio, mobile phone, farmer groups and extension agents. These results as well as those of the descriptive statistics disaggregated by campaign channels (see S1 Table) are indicative that there are systematic differences between participants and non-participants in the FAW campaign and also between participants in the various campaign platforms. These imbalances are subsequently taken care of by the PSM and doubly robust estimators in comparing outcomes between participants and non-participants.

The descriptive statistics for the knowledge questions and scores are reported in Table 2. A large share of the sample farmers responded correctly to most of the FAW knowledge quiz questions, except six questions where the correct responses were below average for both participants and non-participants. The results further indicate that farmers who participated in the campaign significantly outperformed those that were not exposed to the campaign on 25 of the 30 FAW knowledge questions. The results in the lower panel of Table 2 show that relative to non-participants, the campaign participants achieved statistically greater scores on all the four knowledge scores. Overall, the campaign participants on average scored about 12 percentage points higher than non-participants on the knowledge test pertaining to FAW identification, monitoring and management. Disaggregating the knowledge scores by the different campaigns (see S1 Table), we observe statistically significant higher knowledge scores for each of the five campaign options relative to the control group of non-participants. We also notice that, among five campaign options, greater knowledge scores were obtained by farmers who were exposed to both radio and video (i.e., Radio+Video).

**Fig 2:**
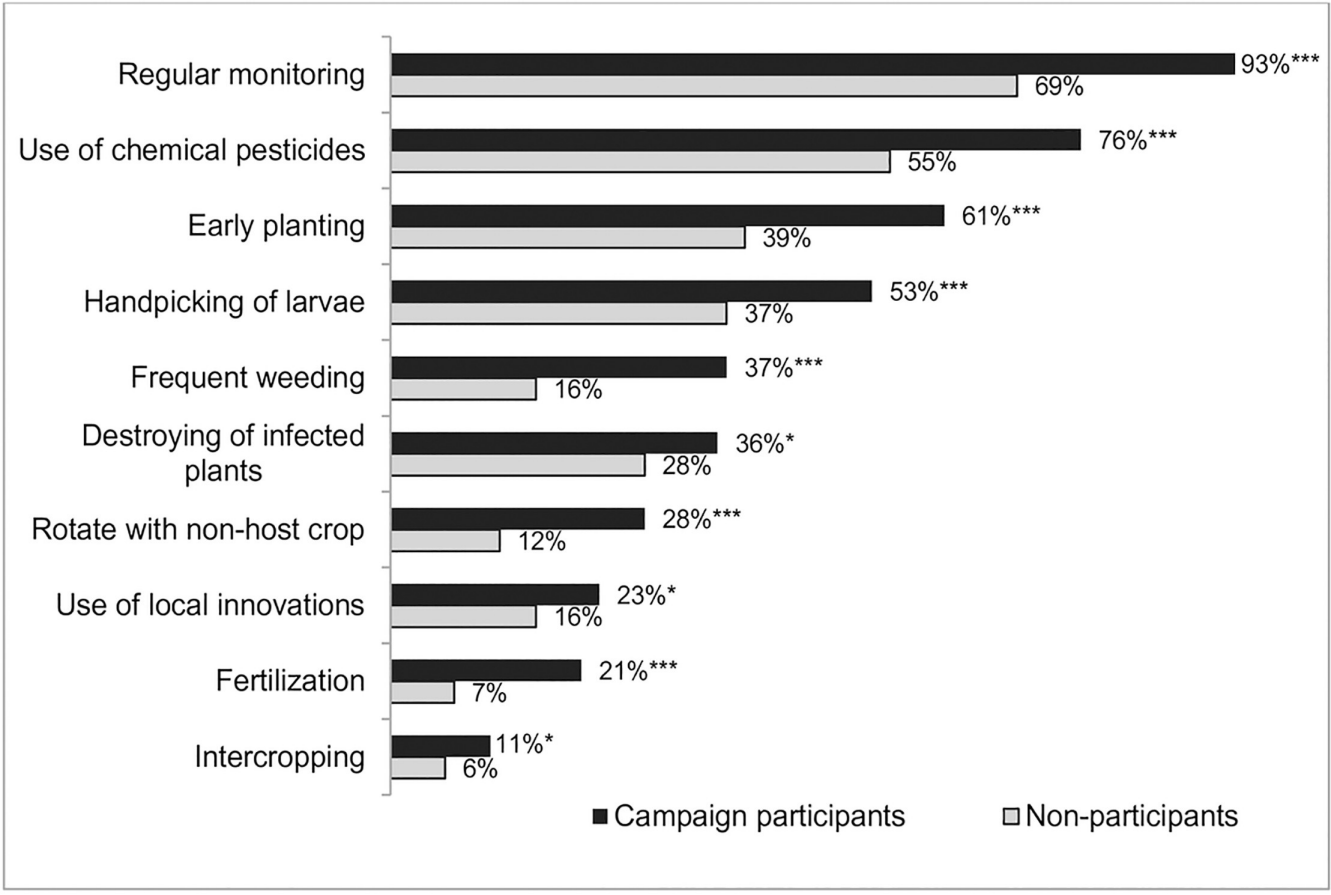
The main FAW management practices implemented by sample households. Fig notes: ***, **, * indicate significant differences between campaign participants and non-participants at the 1%, 5% and 10% significance levels, respectively. [Participants (n = 460; non-participants (n = 147)]. Other FAW management practices that were implemented by very few of the sample farmers include the application of botanical pesticides, trap cropping or planting of repellent plants, and the use of biological control methods.

Fig 2 displays the major agricultural practices and technologies employed by the sample households to prevent, reduce or control FAW infestation in their maize plots. The practices include regular monitoring of maize crop after germination to check for the presence of FAW or damage symptoms; the use of chemical pesticides; handpicking of egg masses and larvae; rotation or intercropping of maize with non-host plants; regular weeding to remove alternative host plants; and local innovations such as applying ash, detergent or soil to maize whorls. Significantly more campaign participants than non-participants applied the various FAW management practices, with discernible differences in terms of the practicing of regular monitoring, early planting and frequent weeding, and the use of chemical pesticides. S1 Table shows that the average number of management practices adopted by the non-participants is three, whereas those implemented by the participants range, on average, from four to seven, depending on the campaign platform.

### Determinants of campaign participation

Table 3 presents the first-stage logistic regression results of the PSM estimator, and this indicates the determinants of participation in any of the campaigns as well as in specific campaigns. For consistency checks, we also report estimates from a multinomial logit model (S2 Table), which is the first-stage results for the doubly robust estimator. Qualitatively similar results were obtained across the two estimation methods. Grouping together the campaign participants and comparing with non-participants, the estimation results in the first column of Table 3 show that the age, gender and education of household heads do not significantly affect campaign participation, suggesting that the FAW campaign was inclusive of both male- and female-headed households, young and older as well as illiterate and educated farmers. Households with higher dependency ratios are less likely to participate in the campaign, perhaps partly because of time pressure on the working-age members in the household.

**Table 3:** Logit estimates for participation in the extension campaign.

	Radio or SMS or Video	Radio only	Video only	Radio+ Video	Radio+ SMS	Radio+SMS +Video
Age	0.003	0.006	0.004	-0.010	-0.073*	-0.008
	(0.009)	(0.010)	(0.015)	(0.014)	(0.038)	(0.029)
Gender	0.069	-0.118	0.682	0.121	-1.008	-0.081
	(0.300)	(0.343)	(0.539)	(0.586)	(0.900)	(1.266)
Education	0.006	0.017	0.017	0.006	-0.072	-0.140
	(0.327)	(0.036)	(0.053)	(0.052)	(0.096)	(0.101)
Household size	-0.018	-0.034	-0.071	0.042	0.127	-0.067
	(0.035)	(0.042)	(0.060)	(0.046)	(0.113)	(0.137)
Dependency ratio	-0.217**	-0.225**	-0.183	-0.365**	-0.189	-0.156
	(0.095)	(0.108)	(0.164)	(0.181)	(0.334)	(0.399)
Land holding	0.003	-0.014	0.007	0.001	-0.010	0.038*
	(0.019)	(0.020)	(0.035)	(0.021)	(0.042)	(0.023)
Input market	0.032	0.054**	-0.016	0.067	0.176**	0.068
	(0.024)	(0.027)	(0.051)	(0.046)	(0.079)	(0.089)
Radio	1.426***	1.827***	0.511	2.696***	2.457**	2.029
	(0.312)	(0.387)	(0.483)	(0.754)	(1.245)	(1.292)
Phone	0.945**	0.663	1.373**	0.785	2.074	—
	(0.367)	(0.426)	(0.638)	(0.675)	(1.349)	
Extension access	0.673**	0.2279	1.283***	1.220***	0.707	1.624**
	(0.281)	(0.331)	(0.412)	(0.383)	(0.737)	(0.716)
Farmer group	0.526*	0.411	0.663	1.167***	1.836***	0.559
	(0.279)	(0.321)	(0.460)	(0.379)	(0.692)	(0.870)
Off-farm activity	0.116	0.201	0.445	0.091	-0.319	0.168
	(0.231)	(0.261)	(0.377)	(0.351)	(0.617)	(0.649)
PPI	-0.029**	-0.018	-0.760***	-0.028	-0.008	-0.038
	(0.013)	(0.015)	(0.023)	(0.020)	(0.033)	(0.038)
Risk preference	0.079**	0.095**	0.080	0.078	0.032	0.077
	(0.039)	(0.044)	(0.071)	(0.062)	(0.119)	(0.131)
District	0.808***	1.084***	-0.723*	0.921**	2.680***	3.233***
	(0.241))	(0.274)	(0.418)	(0.399)	(0.871)	(1.239)
Constant	-0.654	-1.969	-0.024	-3.663***	-5.148**	-3.749
	(0.676)	(0.775)	(1.118	(1.237)	(2.399)	(2.655)
No. of observations	607	373	191	218	155	124

Note

***, **, * denote 1%, 5%, and 10% significance level, respectively.

The probability of participation in the campaign increases with ownership of radio and mobile phones, contact with extension agents and membership in farmer groups. This is unsurprising, given that multiple information channels were used to create widespread awareness of the FAW campaign. The coefficient on the PPI variable is negative and statistically significant, indicating that after controlling for other covariates, poorer households have a higher probability of participating in the campaign. A possible explanation is that agricultural information flows are already biased towards richer households, and this can decrease their likelihood of making efforts to acquire information through, for instance, a video campaign, which appears to be driving this result. Results also indicate that households located in Masindi district have a higher propensity to participate in the campaign than those located in Buliisa. This may be related to geographic proximity to information source, considering that the radio campaign, which generated the most coverage among the three campaign channels, was broadcasted through Radio Kitara, which is situated in Masindi district.

Table 3 also shows some distinct differences in the determining factors for participation in the specific campaign platforms. For instance, ownership of radio significantly enhances exposure to the radio only campaign as well as to the combined exposure to radio and video or radio and SMS campaigns, but not the exposure to video only or all the three campaigns. Conversely, mobile phone ownership is significantly associated with participation in the video only campaign, but not with the video in combination with other campaigns. This is plausible, as households with mobile phones are more likely to be easily contacted to attend a community-based video screening, but those who participated in both the radio and video campaigns could have heard about the video screenings through radio. The results also seem to indicate that households living far from farm input shops are more likely to participate in two of the campaign options (i.e., Radio only and Radio+SMS). This may be suggestive that farmers in close proximity to input suppliers or agro-dealers, which are alternative important sources of agricultural information for smallholder farmers in developing countries [45, 46], have a lower propensity to participate in the FAW campaign.

### Impacts of campaign participation

Before estimating the treatment effects of the FAW campaign, we first check if the common support and covariate balancing conditions are fulfilled. S1 Fig demonstrates that there are sufficient overlaps in the distribution of the propensity scores between participants and non-participants across the various campaign options, suggesting a satisfaction of the common support condition. The covariate balancing test results reported in S3 Table show low pseudo-R^2^, reduced mean bias and insignificant log-likelihood values after matching, and these are all indications of matching quality [34]. We also conducted a matching quality test following Rosenbaum and Rubin [47], and the test results (which can be made available upon request) indicate that in contrast to the unmatched sample, there are no statistically significant differences in covariates between the campaign participants and non-participants after matching, which further confirm the satisfaction of the balancing requirement. S1 Fig also confirms that our matching method is able to balance the distribution of covariates between the campaign participants and non-participants. Overall, these results indicate successful comparability of our treatment and control groups after matching.

#### Impacts of campaign on FAW knowledge scores

Table 4 displays the results of the kernel matching and doubly robust IPWRA estimators of the effects of the FAW campaign participation on knowledge scores and the adoption of management practices. We find that our results are mostly consistent, with the same signs, significance levels and comparable ATT estimates across the two estimation techniques. Looking at the first comparison between campaign participants and non-participants, we find that exposure to the FAW campaign (regardless of the channel) significantly increases farmers’ knowledge of FAW identification, monitoring and management by 22–23%, 15–17% and 18–19%, respectively. This translates into nearly 20% improvement in overall knowledge about FAW among the campaign participants.

**Table 4:** Impacts of the campaign on farmers’ knowledge and management of FAW.

	Kernel matching				Doubly robust estimator		
	ATT	SE	ATT in %	Γ	ATT	SE	ATT in %
***Participants vs*. *non-participants***							
FAW identification score	1.35***	0.26	21.60	4.90–5.00	1.42***	0.24	22.98
FAW monitoring score	0.54***	0.16	14.75	3.40–3.50	0.60***	0.13	16.62
FAW management score	1.64***	0.35	18.30	4.30–4.40	1.67***	0.27	18.72
Overall FAW knowledge score	3.53***	0.63	18.72	6.50–6.60	3.69***	0.53	19.71
Adoption of FAW mgt. practices	1.63***	0.24	53.27	4.90–5.00	1.69***	0.27	55.96
***Radio vs*. *non-participants***							
FAW identification score	1.11***	0.28	17.87	3.10–3.20	1.24***	0.24	20.36
FAW monitoring score	0.39**	0.18	10.54	2.10–2.20	0.50***	0.15	13.81
FAW management score	1.24***	0.36	13.81	2.80–2.90	1.35***	0.29	15.22
Overall FAW knowledge score	2.74***	0.66	14.51	3.50–3.60	3.09***	0.54	16.64
Adoption of FAW mgt. practices	1.39***	0.26	45.72	4.20–4.30	1.49***	0.27	50.00
***Video vs*. *non-participants***							
FAW identification score	1.29***	0.42	20.09	2.90–3.00	0.98***	0.33	15.05
FAW monitoring score	0.31	0.27	8.31	-	0.26	0.19	6.90
FAW management score	1.74***	0.57	19.46	3.10–3.20	1.60***	0.40	17.72
Overall FAW knowledge score	3.34***	1.04	17.50	3.50–3.60	2.84***	0.78	14.71
Adoption of FAW mgt. practices	1.22***	0.41	38.36	2.20–2.30	1.15***	0.33	36.74
***Radio+Video vs*. *non-participants***							
FAW identification score	1.94***	0.26	30.79	9.40–9.50	2.14***	0.30	34.97
FAW monitoring score	0.93***	0.21	25.76	13.2–13.3	0.95***	0.16	26.69
FAW management score	2.52***	0.49	27.80	7.80–7.90	2.71***	0.36	30.80
Overall FAW knowledge score	5.38***	0.87	28.39	16.7–16.8	5.81***	0.68	31.44
Adoption of FAW mgt. practices	2.00***	0.38	63.89	4.40–4.50	2.07***	0.41	68.31
***Radio+SMS vs*. *non-participants***							
FAW identification score	2.16***	0.56	36.61	4.70–4.80	1.69***	0.54	27.80
FAW monitoring score	1.04***	0.32	30.86	3.40–3.50	0.94***	0.28	27.09
FAW management score	2.03***	0.70	23.55	2.70–2.80	2.09***	0.57	24.59
Overall FAW knowledge score	5.22***	1.16	29.18	7.90–8.00	4.72***	1.04	26.15
Adoption of FAW mgt. practices	1.83***	0.69	73.86	2.10–2.20	2.26***	0.69	73.62
***Radio+SMS+Video vs*. *non-participants***							
FAW identification score	2.15***	0.52	36.26	5.30–5.40	1.89***	0.42	30.19
FAW monitoring score	1.10***	0.26	32.07	3.40–3.50	0.74***	0.25	20.27
FAW management score	2.63***	0.70	31.72	3.80–3.90	2.04***	0.47	22.97
Overall FAW knowledge score	5.88***	1.04	32.30	5.30–5.40	4.66***	0.87	24.83
Adoption of FAW mgt. practices	2.97***	0.66	98.02	6.10–6.20	3.32***	0.70	103.43

Notes

*** and ** denote 1% and 5% significance level, respectively.

Adoption of FAW mgt. practices = the number of FAW management practices adopted by a household. Γ = Critical level of hidden bias.

Table 4 also reveals interesting heterogeneous effects of the campaign on FAW knowledge, when we disaggregate the analysis by campaign channel. We find that exposure to multiple campaigns generates greater impacts on FAW knowledge than exposure to a single campaign. The ATT estimates for the single campaigns range from 15–18%, while those of the multiple campaigns vary from 28–32%. We also find that while exposure to only radio or video campaign is associated with a 15–18% increase in FAW knowledge, combined exposure to radio and video channels (Radio+Video) builds overall FAW knowledge of about 28–31%. Thus, the combined effect of radio and video campaigns on FAW knowledge is almost double the effect size of participating in either of the two campaigns alone, pointing to an additive effect of the two channels. Furthermore, we see that exposure to all the three campaign channels increases overall knowledge score by 25–32%, suggesting that there is little to no enhanced effect of adding SMS to a Radio+Video campaign if the goal is to increase farmers’ knowledge of FAW.

#### Impacts of campaign on the adoption of FAW management practices

The results in Table 4 further indicate that exposure to any of the campaign channels exerts a positive and significant effect on the adoption of FAW management practices. In particular, the campaign significantly increases the number of FAW management practices employed by the participants relative to non-participants by two practices or about 53–55%. Disaggregating by campaign options, here again we find that the magnitude of the effects of participation in multiple campaigns on the adoption of FAW management practices is greater than the effect size of participating in a single campaign. More specifically, exposure to a single campaign (i.e., Radio only or Video only) enhances the adoption of FAW management strategies by one extra practice, whereas exposure to two campaign channels (i.e., Radio+Video or Radio+SMS) triggers the uptake of two extra practices relative to households who were not exposed to the campaign. The results also indicate that in contrast to the knowledge outcome analysis above, households exposed to all three campaign channels adopted the highest number of FAW management techniques of three extra practices relative to the unexposed group. Taken together, these results imply that there is an additive effect of the campaign channels on the adoption of FAW management practices, in that exposure to increasing numbers of channels corresponds to increased uptake of practices for FAW management.

S4 Table presents the heterogeneous effects of the campaign on the adoption of specific FAW management strategies. For instance, the kernel matching results show that relative to non-participants, farmers who participated in the campaign are 33% more likely to regularly monitor their maize to check for presence of FAW; 67% more likely to handpick and crush egg masses and larvae; and 124% more likely to constantly weed their farms to remove alternative host plants for the FAW. The results also show that among the FAW managements practices, the lowest ATT of about 15% is observed for the use of synthetic pesticides, and it is even not statistically significant in the case of kernel matching or weakly significant (at 10% level) when using the double robust estimator. This may be suggestive that the campaign did not significantly enhance the use of chemical pesticides, which is compelling given the known negative impacts of chemical pesticides on human health and the environment. As noted earlier, the campaign was designed along the principles of IPM, including topics on hazards and safe use of pesticides, and this may have contributed to this finding.

#### Robustness checks

As mentioned earlier, the reliability of our impact estimates using PSM depends on selection on observables, and thus our results would be biased if there is unobserved heterogeneity (hidden bias) between the campaign participants and non-participants that affect our outcomes of interest. We check the sensitivity of the ATT estimates to unobserved heterogeneity by computing the Rosenbaum bounds (critical gamma levels, Γ), which measure how large the difference in unobserved variables that influence the decision to participate in the campaign would have to be in order to affect the estimated impacts. In general, the results of the critical gamma levels (Γ) in Table 4 suggest that the observed positive and significant impacts of the FAW campaign are robust to unobservables or hidden bias. For instance, the Γ value of 4.90ꟷ5.00 in the upper part of Table 4 implies that the significant effect of campaign participation on FAW identification or adoption of management practices would be questionable only if households with the same covariates differ in their odds of participating in the campaign by a factor of 390ꟷ400%.

We also test the robustness of our ATT results using two alternative PSM estimatorsꟷ nearest neighbour (NN) matching and radius matching with a calliper of 0.05. Detailed descriptions and discussions on the advantages and disadvantages of the different matching algorithms can be found in [34]. The results are presented in S5 Table. The results from these two algorithms are fairly similar to those of the kernel matching and the doubly robust estimator, signifying that our impact estimates are robust and not sensitive to the matching method employed. For example, participation in the campaign increases overall FAW knowledge (the adoption of FAW management practices) by 17% (53%), 19% (53%), 20% (56%) and 21% (56%) using Radius matching, Kernel matching, doubly robust estimator and NN matching, respectively.

#### Incremental impacts of other campaign channels over radio

So far we have shown that households that participated in any of the campaigns outperformed the non-participants in knowledge about FAW and the adoption of management practices. An important follow-up question then is how do the different campaigns compare against each other? We address this question by restricting our sample to households who participated in the campaign, and assess the incremental impact of the campaigns over the radio only campaign, which is the common campaign option. By limiting the sample to campaign participants, we reduce bias due to unobserved heterogeneity in the propensity to participate in the campaign.

Table 5 shows the kernel matching and the doubly robust estimates of the impacts of the campaigns relative to households exposed to only the radio campaign. Once again we find generally consistent results across the two estimators. Results show that households that participated in only the video campaign significantly increased their ability to identify, monitor and manage FAW compared to those who participated in only the radio campaign. A number of reasons could explain this observation. First, radio’s lack of visual imagery limits the possibility to see some features and damage symptoms of the pest. Secondly, although the radio campaign was interactive with call-in options, not all farmers are able to phone in to ask for clarifications. A video screening, on the other hand, allows face-to-face oral and visual communication, which is appropriate when communicating complex messages, such as the adoption of IPM practices [48]. Moreover, a video screening has a facilitator who can clarify issues on the spot. Finally, video screenings provide opportunity for social interactions, which can stimulate peer learning and support even after the campaign has ended.

**Table 5:** Impact estimates using radio campaign as the control group.

	Kernel matching				Doubly robust estimator		
	ATT	SE	ATT in %	Γ	ATT	SE	ATT in %
***Video only vs*. *Radio only***							
FAW identification score	0.67**	0.30	9.25	1.80–1.90	0.55**	0.27	7.54
FAW monitoring score	0.32*	0.19	8.27	1.50–1.60	0.40***	0.11	9.83
FAW management score	1.31***	0.45	13.94	3.10–3.20	0.96***	0.37	10.03
Overall FAW knowledge score	2.30***	0.72	11.21	3.10–3.20	1.53**	0.64	7.42
Adoption of FAW mgt. practices	0.31	0.36	7.29	—	0.06	0.32	1.43
***Radio+Video vs*. *Radio only***							
FAW identification score	0.78***	0.20	10.48	2.40–2.50	0.91***	0.19	12.36
FAW monitoring score	0.41***	0.10	9.98	2.20–2.30	0.42***	0.09	10.24
FAW management score	1.30***	0.30	12.65	3.20–3.30	1.26***	0.27	12.29
Overall FAW knowledge score	2.50***	0.47	11.45	3.70–3.80	2.59***	0.41	11.94
Adoption of FAW mgt. practices	0.68**	0.29	15.35	1.40–1.50	0.68**	0.29	15.11
***Radio+SMS vs*. *Radio only***							
FAW identification score	0.41	0.42	5.66	—	0.39	0.37	5.28
FAW monitoring score	0.13	0.18	3.10	—	0.29*	0.16	7.04
FAW management score	-0.26	0.41	-2.50	—	0.18	0.43	1.73
Overall FAW knowledge score	0.29	0.57	1.32	—	0.86	0.64	3.93
Adoption of FAW mgt. practices	0.83	0.64	19.12	—	1.06*	0.60	24.82
***Radio+SMS+Video vs*. *Radio only***							
FAW identification score	0.62*	0.37	8.40	1.80–1.90	0.67*	0.38	8.97
FAW monitoring score	0.33*	0.17	7.97	1.10–1.20	0.17	0.18	4.04
FAW management score	0.35	0.56	3.35	—	0.08	0.41	0.74
Overall FAW knowledge score	1.30*	0.78	5.93	1.40–1.50	0.92	0.74	4.09
Adoption of FAW mgt. practices	1.31**	0.54	27.93	2.70–2.80	1.57***	0.57	31.65

Note

*** denotes 1% significance level.

Adoption of FAW mgt. practices = the number of FAW management practices adopted by a household. Γ = Critical level of hidden bias.

Results further indicate that when households are exposed to both radio and video, it leads to significant increases in both FAW knowledge and the implementation of management practices. Conversely, we find no robust significant effects of exposure to both radio and SMS over radio alone. We also find that concurrent exposure to all three channels produces the highest effect on the adoption of management practices, and this is likely to be driven by the video campaign, given that the SMS campaign did not exhibit significant incremental effect over the radio campaign. Taken together, these results suggest that among the three channels, video was the most effective in terms of achieving the goals of the campaign; however, it achieves greater impacts when combined with radio.

## Conclusions

This article explored the effects of ICT-based extension services on farmers’ knowledge and management of FAW, a serious invasive pest of maize in Africa. The study is based on an intervention in western Uganda that used three complementary ICT-based channels (i.e., interactive radio, mobile SMS messages and village-based video screenings) with the aims of increasing farmers’ awareness on the identification, prevention and sustainable management of FAW, and improving farmers’ practices in managing FAW on their farms.

Using the doubly robust and various propensity score matching estimators, we found consistent evidence that participation in the ICT-based extension campaigns significantly increased farmers’ knowledge about FAW and stimulated the adoption of agricultural technologies and practices for the management of the pest. Even the most conservative estimates suggest that the campaign contributed to a 17% increase in knowledge related to FAW identification, monitoring and management, which translated into the adoption of two additional FAW management practices. The sensitivity analysis of hidden bias shows that our results are not likely driven by unobservable household characteristics. When analysing the unique and combined effects of the three ICT-based channels, we found that exposure to multiple campaign channels leads to higher impacts on FAW knowledge scores and the adoption of management practices than exposure to a single campaign. We also found differential outcomes between the three channels. For example, radio proved most effective in achieving widespread coverage; and while the observed impacts on FAW knowledge and management appear to be considerably driven by video, greater gains are achieved when it is complemented by radio.

Overall, our findings imply that in the ongoing battle against FAW in SSA, ICT-based extension campaigns hold great potential to improve farmers’ knowledge on how to identify, monitor and manage the pest, which, in turn, can trigger the adoption of complementary management strategies. The results also imply that to achieve higher awareness and knowledge of FAW that will also translate into behavioural changes, using complementary ICT channels that repeat and reinforce messages is worthwhile. However, in a low-literacy population, as in our case, ICT applications that allow both verbal and visual communication (such as village-based video screenings) should be prioritised when an array of channels are being considered. Our data did not permit analysis of the unique effects of SMS, but the results generally point to a weak to no impact of SMS relative to the other two campaign channels. The SMS campaign under study relied mostly on push-based messages, which has the risk of being seen as spam [12]. Moreover, text-based SMS is considered to be less suitable for illiterate farmers and is also limited in the amount and type of content that can be delivered [49]. Thus, future implementations of mobile–based extension campaigns in similar environments should consider alternative models such as pull SMS and voice-based services, coupled with a better understanding of the local settings.

A limitation of this study is that we have relied on cross-sectional data. While we have tried to control for observable confounding factors using various selection-on-observables estimators and also confirmed that our results are robust to hidden bias using Rosenbaum bounds sensitivity analysis, further research involving experimental or panel data designs would be needed to properly account for potential bias from unobserved heterogeneity and to test our findings. Moreover, there is a possibility of positive FAW knowledge spillovers from the campaign participants to non-participants, and thus the exact magnitudes of the estimated effects of campaign participation relative to non-participants could even be larger than those reported. Finally, we have focused on the role of complementary ICT-enabled extension services in improving farmers’ knowledge and management of FAW. It would also be interesting to study the complementarities between ICT-based and conventional extension approaches such as farmer training and demonstrations in achieving the outcomes of interest, particularly in the current context in Uganda where agricultural extension services have been reformed and extension-farmer ratios are improving [50], and we leave this for future research.

## Supporting information

S1 File Questionnaire. (DOCX)

S2 File Data. (CSV)

S1 Table Descriptive statistics by FAW campaign channel (see Table 1 for the description of the covariates). (DOCX)

S2 Table Multinomial logit estimates for participation in the FAW extension campaign. (DOCX)

S3 Table Balancing tests before and after kernel matching. (DOCX)

S4 Table Campaign effects on the adoption of specific FAW management practices. (DOCX)

S5 Table Impact estimates using alternative matching algorithms. (DOCX)

S1 Fig Kernel density distribution showing overlap and balancing between participants and non-participants of the extension campaign channels. (TIFF)
