# Supplementary material for: The impact of ICT-enabled extension campaign on farmers’ knowledge and management of fall armyworm in Uganda
Source: PLoS One. 2019 Aug 21;14(8):e0220844. doi: 10.1371/journal.pone.0220844 (PMC6703685; doi:10.1371/journal.pone.0220844)
Supplement: S1 File — (DOCX) [file pone.0220844.s001.docx]

**Survey on Farmers’ Knowledge, Attitudes and Practices Related to Fall Armyworm in Uganda**

| “Good morning/afternoon. We are coming from CABI with permission from the local government. We are conducting a survey looking at farmers’ pest management practices. We would like to ask you some questions that should take about one hour of your time. We would like to share some of this information widely in order that more people understand what practices farmers implement and the way they implement them.  We would like to interview farmers that make the decisions on crop management.  Your name will not appear in any data that is made publicly available. The information you provide will be used purely for research purposes; your answers will not affect any benefits or subsidies you may receive now or in the future. Do you consent to be part of this study?”  **Does the household consent to provide information Yes ( ), NO ( ).**  **If NO, do not continue with the interview** |
| --- |
|  |
|  |

**Section 1 – Data Handlers**

| Date (dd/mm/yyyy) |  |
| --- | --- |
| Enumerator name *(select your name)* |  |
| Supervisor name *(select supervisor name)* |  |
| District | 1= Buliisa 2=Masindi 3=Kiryandongo |
| Sub-county | 1=Kihungya 2=Kigwera 3=Ngwedo  4=Butiaba 5= Pakanyi 6=Bwijanga 7=Miirya 8=Kimengo 9=Kigumba |
| Village |  |
| GPS coordinates (UTM) (*if internet connectivity is low it would be possible to collect this information at the end of the survey)* | N: _____________GPSN  S: _____________GPSS  E: _____________GPSE  W: _____________GPSW |

**Section 2: Household Respondent and Type**

*Ideal respondent: household head and/or spouse. Most of these questions can be completed without having to question the respondent directly (e.g. gender). Be sensitive about the way you gather this information.*

2.1 Did your household cultivate maize during **the last cropping season** (April to July 2018)?

1. Yes [ ]
2. No [ ]

**If answer to question 2.1 is no, do not continue with the rest of the survey.**

2.2 Name of (main respondent):

- 1. First name ___________________________________
  2. Family name______________________________________
  3. Phone number (1)__________________(2) __________________

2.3 Gender of the respondent:

1. [ ] Male

2. [ ] Female

2.4 What is the relationship of the respondent to the head of household?

1[ ] Head 2[ ] Spouse 3 [ ] Parent 4[ ] Child 5[ ] Other family member 6[ ] Unrelated

**Section 3: Household* composition and characteristics**

** A household includes all persons who typically eat from the same pot for the majority of the year (six months and above). Members who live somewhere else and only come to visit for a period of less than six months are not to be considered household members.*

3.1 How many persons are in your household, including yourself?

[ ]

3.2 How many household members are in the following age group, including yourself?

| **Age bracket** | **Number of HH members** | |
| --- | --- | --- |
|  | **Male** | **Female** |
| 0-14 years |  |  |
| 15-64 years |  |  |
| Above 64 years |  |  |

Note: Enumerators should make sure that the numbers in 3.2 add up to that in 3.1.

- 1. Are all household members aged 6 to 12 currently in school?

1. Yes [ ]
2. No [ ]

3.4 How many household members work on the farm?

[ ]

3.5 How many household members are involved in off-farm income-generating activity, such as non-farm business, casual labour, civil service job, artisan work, etc.

[ ]

- 1. Information about household head and spouse

| **HH ID** | **Name** | **Age** | **Gender** 1=Male  2=Female | **Working on the farm?**  0=no  1=yes, at times (part-time)  2=yes, farming is main occupation (full-time) [2] | **Highest level of education attained**  0= none  1=primary  2=secondary (O-level)  3=secondary (A-level)  4=vocational  5= university | **Number of years of schooling**  *(Do not count years in nursery school)* |
| --- | --- | --- | --- | --- | --- | --- |
| Head |  |  |  |  |  |  |
| Spouse |  |  |  |  |  |  |

3.7 What it the highest level of education attained by the most educated person in the household?

0= none 1= primary school 2= secondary (O-level) 3= secondary (A-level) 4= vocational 5=university

3.8 How many years has the household head been living in this village?

[ ]

**Section 4: Participation in FAW campaign**

- 1. During the past cropping season (April to July 2018), did anyone in your household hear about the FAW pest through RADIO?

1. Yes [ ]
2. No [ ]

4.1a If yes, who in the household received the radio messages on FAW?

(*select one answer*)

[ ] Head [ ] Spouse [ ] Child [ ] Both head and spouse [ ] Head, spouse and child

[ ] Other household member

4.1b If yes, the FAW messages were broadcasted on which radio station?

[ ]

4.1c If yes, how many FAW radio episodes did the household member listen to during the past cropping season?

[ ]

- 1. During the past cropping season (April to July 2018), did anyone in your household receive FAW messages through SMS

1. Yes [ ]
2. No [ ]

4.2a If yes, who in the household received the FAW messages via SMS?

(*select one answer*)

[ ] Head [ ] Spouse [ ] Child [ ] Both head and spouse [ ] Head, spouse and child

[ ] Other household member

4.2b If yes, from which SMS source did the household member receive the FAW messages?

1= Hamwe mFarmer Platform 2=USSD code *180# 3= SMS ‘FAW’ to 6565 4=Other (please specify)

4.2c How many FAW messages did the household member receive via SMS during the past cropping season?

[ ]

4.3 During the past cropping season (April to July 2018), did anyone in your household watch a video about FAW through the Kibanda Boda or CrowdPullerz public video screenings? (*Explain and show pictures of Kibanda Boda and CrowdPullerz screenings to respondents*).

1. Yes [ ] continue
2. No [ ] continue

4.3a If yes, who in the household participated in the FAW video screenings?

(*select one answer*)

[ ] Head [ ] Spouse [ ] Child [ ] Both head and spouse [ ] Head, spouse and child

[ ] Other household member

4.3b If yes, on which platform did the household member watch the video on FAW?

1=Kibanda Boda screenings 2= CrowdPullerz public video screenings 3. Both

IF YES TO 4.1 OR 4.2 OR 4.3:

4.4 In general, how useful did your household find the information on FAW?

1=not at all useful 2=a little useful 3=somewhat useful 4=very useful

4.5 During the past cropping season, did your household implement the knowledge gained from the FAW campaign?

1. Yes, fully 2. Yes, partially 3. No

4.6 If you implemented partially or did not implement the gained knowledge, why?

1. [ ] Did not understand the message

2. [ ] The message came too late

3. [ ] Lack of money to implement

4. [ ] No FAW attack during the past cropping season

5. [ ] FAW infestation was not severe to warrant actions

6. [ ] Other specify

4.7 (a) Did you share with other farmers the advice you received on FAW?

1. Yes [ ]
2. No [ ]

4.8 (b) If yes, how many farmers did you share with?

[ ]

4.9 Apart from radio, SMS and video screenings, did your household use the following sources for information on FAW in the past cropping season?

| **Source** | 1=Yes 2=No |
| --- | --- |
| Own experience |  |
| Extension agents |  |
| Neighbours/Friends/Relatives |  |
| Demonstration plots/ field days/ farmer field school |  |
| Farmer group |  |
| Agro dealers |  |
| Plant clinic /plant doctor |  |
| NGO workers |  |
| Others (specify) |  |

**Section 5: Knowledge and attitude toward FAW**

| Now I would like to ask you some questions about the Fall Armyworm pest (*Add local name*). Please respond to each of the questions below by indicating whether you **agree, disagree** or you **don’t know.** | |
| --- | --- |
| **Awareness and identification** | |
| Q1. FAW attacks ONLY maize and not other crops. | 1=Agree  2=Disagree  3=Don’t know |
| Q2. The FAW pest comes from seeds or is spread through seeds. | 1=Agree  2=Disagree  3=Don’t know |
| Q3. If not attended to, FAW can cause 100% loss of maize yield. | 1=Agree  2=Disagree  3=Don’t know |
| Q4. FAW pest attack all stages of maize growth, from seedlings to cob feeling stages. | 1=Agree  2=Disagree  3=Don’t know |
| Q5. Adult FAW is a moth that lays creamish white to grey masses of eggs on maize leaves. | 1=Agree  2=Disagree  3=Don’t know |
| Q6. After 2-3 days, eggs hatch into caterpillars that are green in colour and turn brown to black as they grow. | 1=Agree  2=Disagree  3=Don’t know |
| Q7. Older caterpillars have an upside-down Y-shaped mark on forehead. | 1=Agree  2=Disagree  3=Don’t know |
| Q8. Older caterpillars have four dark spots forming a square on the second to last segment from the tail. | 1=Agree  2=Disagree  3=Don’t know |
| Q9. Signs of FAW damage include small pinhole (scratches) and large window like holes with irregular edges on maize leaves. | 1=Agree  2=Disagree  3=Don’t know |
| Q10. Heavily infested plants have moist sawdust like material (frass) on stems, leaves, and in the funnels of the maize plants. | 1=Agree  2=Disagree  3=Don’t know |
| **Monitoring** |  |
| Q11. Early detection of the FAW allows early control that will help minimise damage. | 1=Agree  2=Disagree  3=Don’t know |
| Q12. It is important to visit your maize farm 2-3 weeks after planting to check for the presence of FAW. | 1=Agree  2=Disagree  3=Don’t know |
| Q13. It is important to continue monitoring your farm every 3 days for signs of FAW. | 1=Agree  2=Disagree  3=Don’t know |
| Q14. To monitor FAW, walk along the edges of the maize fields. | 1=Agree  2=Disagree  3=Don’t know |
| Q15. To monitor FAW, examine 50 maize plants per acre or 10 maize plants in 5 different locations in the maize farm, and record the number of maize plants with FAW infestation. | 1=Agree  2=Disagree  3=Don’t know |
| **Management practices** |  |
| Q16. On small-scale farms, hand picking and crushing egg masses and caterpillars can be a cheap, safe and effective measure of control. | 1=Agree  2=Disagree  3=Don’t know |
| Q17. Take action to control FAW when 10 or more out of 50 randomly selected maize plants in your field have signs of FAW. | 1=Agree  2=Disagree  3=Don’t know |
| Q18. Early planting at the onset of the rain can prevent or reduce FAW infestation. | 1=Agree  2=Disagree  3=Don’t know |
| Q19. Crop rotation and intercropping with crops such as beans, pigeon pea and cassava can help to reduce FAW infestation. | 1=Agree  2=Disagree  3=Don’t know |
| Q20. Regular weeding of farm can help prevent FAW infestation. | 1=Agree  2=Disagree  3=Don’t know |
| Q21. Chemical pesticides for managing FAW are NOT dangerous to humans. | 1=Agree  2=Disagree  3=Don’t know |
| Q22. It is important to use protective gear when mixing or spraying pesticides. | 1=Agree  2=Disagree  3=Don’t know |
| Q23. Spray pesticides when there are signs of rain. | 1=Agree  2=Disagree  3=Don’t know |
| Q24. Spray pesticides between 6 to 10 am or from 4 to 7pm when the FAW is very active | 1=Agree  2=Disagree  3=Don’t know |
| Q25. Empty pesticide containers can be reused for other purposes. | 1=Agree  2=Disagree  3=Don’t know |
| Q26. To control FAW, do not spray pesticide to the whole maize plant. Spray only into the maize funnel. | 1=Agree  2=Disagree  3=Don’t know |
| Q27. Do not spray pesticides when maize is mature and the cob is drying. | 1=Agree  2=Disagree  3=Don’t know |
| Q28. Mix different pesticides to make them more effective in controlling FAW. | 1=Agree  2=Disagree  3=Don’t know |
| Q29. Apply pesticide at a dose of about 20ml to 20 litres of water for small caterpillars. | 1=Agree  2=Disagree  3=Don’t know |
| Q30. After spraying, monitor after 4 days to see if the FAW are dead. If not dead, re-spray affected plants within 7-14 days. | 1=Agree  2=Disagree  3=Don’t know |

**Section 6: Farmers’ management practices against FAW**

6.1 Have you seen this pest on your maize farm **last year**?

*Show to the farmer pictures illustrating presence of FAW at different reproductive stages*

1. Yes [ ]
2. No [ ]

6.2 Did you see the same pest on your maize during **the last cropping season** (April to July 2018)?

1. Yes [ ]
2. No [ ]

6.3 Apart from maize, which other crops were attacked by the FAW pest (maximum 3)

1[ ] 2[ ] 3[ ]

6.4 During the last cropping season (April to July 2018), did you start monitoring your maize after germination and continuously visited your maize farm to check for signs of the FAW pest?

1. Yes [ ]
2. No [ ]

6.5 During the last cropping season (April to July 2018), did you record the level of FAW infestation before deciding on when and what FAW control measure to implement?

1. Yes [ ]
2. No [ ]

6.6 During the last cropping season (April to July 2018), did you implement any of the following practices to **prevent** FAW from infesting your maize plants?

| **Intervention** | **1=Yes 2 =No** |
| --- | --- |
| 1. Planting of maize at the same time at the onset of the rains (early planting) |  |
| 2. Crop rotation: alternate maize with crops that are not attacked by FAW |  |
| 3. Intercrop with less susceptible crops such as beans and cassava |  |
| 4. Regular weeding of maize fields and surrounding areas |  |
| 5. Add manure or chemical fertilizer for strong maize plants that can withstand FAW damage and compensate for damage done |  |
| 6. Planting resistant maize varieties |  |

6.7 If yes to 6.2, did you implement any of the following practices to **control** the FAW during the past cropping season?

| **Intervention** | **1=Yes 2 =No** |
| --- | --- |
| 1. Application of ash on caterpillars |  |
| 2. Trap cropping or planting repellent plants e.g. push-pull technology |  |
| 3. Application of chemical pesticides |  |
| 4. Application of botanical (plant-based) pesticides e.g. neem, pepper, tephrosia |  |
| 5. Handpicking egg masses and caterpillars |  |
| 6. Uprooting and burning of infected plants |  |
| 7. Biological control methods (encouraging natural enemies into maize field) |  |
| 8. Application of detergents or soaps |  |
| 9. Other (please specify) |  |

6.7 For each of the FAW preventive and control practices (in 6.6. and 6.7) that your household implemented, do you think they were successful?

1= Extremely successful 2=Somewhat successful 3=Not successful

**Section 7: Maize production**

7.1 What is the unit that you use to measure farm size?

1[ _ ] Hectares 2[ _ ] Acres 3[ _ ] Ares 4[ _ ] Other (specify_____________)

7.2 Land holding

|  | Size |
| --- | --- |
| 1. Land owned by household with ownership rights |  |
| 1. Not owned, rented in |  |
| 1. Not owned, borrowed or sharecropped in, etc. |  |
| 1. Amount of land available to household for all activities (1+2+3) |  |

7.3 During the most recent cropping season, what was the total area cultivated with maize?

[ ] *Unit will appear automatically*

7.4 During the last cropping season, how many plots did you grow maize on?

[ ]

7.5 Number of years household has been growing maize?

[ ]

7.6. Who in the household makes the decision on maize production (e.g. what to grow, area, inputs etc.)?

1=Household head 2=Spouse 3= Head & spouse jointly

4=Other male household member 5=Other female household member 6=Other (specify)

**Section 8: Shocks**

8.1 In the past cropping season, have the household’s agricultural production been affected by the following stresses or shocks?

| *Stress/shock* | *1=Yes 2=No* |
| --- | --- |
| Drought or insufficient water |  |
| Excess rain or flood |  |
| Pests/diseases (**other than FAW**) |  |
| Crop destroyed by livestock |  |
| Sickness |  |
| Strong wind |  |

**Section 9: Household assets**

9.1 How many, if any, livestock has your household currently?

*Fill in 0 if none*

| *Livestock type* | *Number* |
| --- | --- |
| Cattle (bulls, cows, steers, heifers, calves, oxen) |  |
| Pigs |  |
| Goats |  |
| Sheep |  |
| Chicken and ducks |  |
| Other |  |

9.2 Does your household own any of the following items?

*Fill in 0 if none*

| *Assets* | *Number* | *Assets* | *Number* |
| --- | --- | --- | --- |
| Radio |  | Hoe |  |
| Television |  | Tractor |  |
| Car/vehicle |  | Sprayer |  |
| Motor cycle |  | Other.................... |  |
| Bicycle |  | Other.................... |  |
| Refrigerator |  | Other.................... |  |
| Mobile phone |  | Other.................... |  |

9.3 What is the main material used for roofing the house (main building)?

1 [ _ ] Thatch or tins

2 [ _ ] Iron sheets, concrete, tiles, asbestos, or other

9.4 What is the main building material used for the walls of the house (main building)

1 [ _ ] Unburnt bricks with mud, mud and poles, or other

2 [ _ ] Unburnt bricks with cement, wood, tin/iron sheets, concrete/stones, burnt stabilized bricks, or cement blocks

9.5 What source of energy does the household mainly use for cooking?

1 [ _ ] Firewood, cow dung or grass

2 [ _ ] Charcoal, paraffin stove, gas, biogas, electricity (regardless of source), or other

9.6 What type of toilet facility does the household mainly use?

1 [ _ ] No facility/bush/polythene bags/bucket/etc., or other

2 [ _ ] Uncovered pit latrine (with or without slab), Ecosan (compost toilet), or covered pit latrine without slab

3 [ _ ] Covered pit latrine with slab

4 [ _ ] VIP latrine, or flush toilet

9.7 Does every member of the household have at least one pair of shoes?

1. Yes [ ]
2. No [ ]

**Section 10: Access to institutional support services**

10.1 Did you or any of your household members, use any of the following sources of credit in the past 12 months?

[ _ ] Formal loans (bank) 1=Yes; 2=No

[ _ ] Small loans through micro-credit 1=Yes; 2=No

[ _ ] Informal loans (from family members or friends) 1=Yes; 2=No

[ _ ] Credit from input providers/ traders / buyer of harvest 1=Yes; 2=No

[ _ ] Credit from cooperatives /associations 1=Yes; 2=No

10.2 Did you or any member of the household receive any subsidized or free input during the last season?

1. Yes [ ]
2. No [ ]

10.3 If yes, which input(s)?

1= Seed 2=Fertilizer 3=Pesticide 4=Seed and Fertilizer 5=Seed and Pesticide 6 =Fertilizer and Pesticide 7= Seed, Fertilizer and Pesticide 8=Other (please specify)

10.4 If yes, from which source?

1= Government 2=Neighbours/fellow farmer 3=Family member 4=Other

10.5 In the past cropping season, how many contacts did your household have with an extension officer for agricultural information?

[ ]

10.6

| **Distance from home to the nearest** | | **Km** |
| --- | --- | --- |
| 1 | All-weather road |  |
| 2 | Farm input shop |  |
| 3 | Market in case you want to sell your farm products |  |
| 4 | Nearest extension office |  |

**Section 11: Social capital, risk attitude and trust**

12.1a Do you or anyone in your household belong to a farmer group or association?

1. Yes [ ]
2. No [ ]

12.1b If yes, how many times did the household member participate in activities or meetings of the farmer group in the past 12 months?

[ ]

12.2 How do you see yourself: are you generally a person who is fully prepared to take risks or do you try to avoid taking risks? Please choose a number of a scale between 0 and 10,

where 0 **means not at all willing to take risks** and **10 means very willing to take risks**

0 1 2 3 4 5 6 7 8 9 10

**End – Thank you very much for your time. The information provided which will be treated confidentially**
