# Supplementary material for: The impact of ICT-enabled extension campaign on farmers’ knowledge and management of fall armyworm in Uganda
Source: PLoS One. 2019 Aug 21;14(8):e0220844. doi: 10.1371/journal.pone.0220844 (PMC6703685; doi:10.1371/journal.pone.0220844)
Supplement: S1 Table — (DOCX) [file pone.0220844.s003.docx]

S1 Table.

|  | Radio only | Video only | Radio+  Video | Radio+ SMS | Radio+SMS  +Video | Non-participants |
| --- | --- | --- | --- | --- | --- | --- |
| *Covariates* |  |  |  |  |  |  |
| Age | 43.93 | 42.60 | 43.20 | 37.95* | 40.52 | 43.46 |
| Gender | 0.84 | 0.88 | 0.90** | 0.86 | 0.95* | 0.79 |
| Education | 7.75*** | 6.86 | 7.44 | 7.50 | 8.05 | 6.62 |
| Household size | 6.43 | 7.34 | 7.42 | 6.86 | 5.90 | 6.99 |
| Dependency ratio | 1.28** | 1.57 | 1.29* | 1.54 | 1.05* | 1.58 |
| Land holding | 3.31 | 2.49 | 4.55* | 3.18 | 11.62*** | 2.66 |
| Input market | 4.48 | 3.25 | 3.98 | 3.79 | 3.30 | 3.40 |
| Radio | 0.94*** | 0.71 | 0.97*** | 0.95*** | 0.95*** | 0.66 |
| Phone | 0.91*** | 0.91** | 0.92*** | 0.95* | 1.00** | 0.79 |
| Extension access | 0.23 | 0.45*** | 0.42*** | 0.41*** | 0.52*** | 0.16 |
| Farmer group | 0.26*** | 0.34*** | 0.41*** | 0.55*** | 0.24 | 0.16 |
| Off-farm activity | 0.52** | 0.52 | 0.52 | 0.41 | 0.52 | 0.41 |
| PPI | 49.84*** | 41.54 | 48.77*** | 50.05** | 54.14 | 43.58 |
| Risk preference | 5.27** | 4.74 | 5.73*** | 5.36 | 5.05 | 4.55 |
| District | 0.77*** | 0.32*** | 0.71*** | 0.86*** | 0.86*** | 0.54 |
| *Outcome variables* |  |  |  |  |  |  |
| FAW identification score | 73.30*** | 74.90*** | 82.70*** | 77.70*** | 81.40*** | 62.52 |
| FAW monitoring score | 82.20*** | 80.60** | 90.40*** | 88.20*** | 87.60** | 72.60 |
| FAW management score | 68.07*** | 70.87*** | 78.73*** | 70.60*** | 72.67*** | 59.13 |
| Overall FAW knowledge score | 72.20*** | 73.83*** | 80.97*** | 74.23*** | 78.10*** | 62.53 |
| Adoption of FAW mgt. practices | 4.50*** | 4.28*** | 5.11*** | 5.33*** | 6.53*** | 2.95 |
| *Number of observations* | 255 | 65 | 93 | 22 | 21 | 147 |

Note: ***, **, * indicate significant differences between participants of the various campaign channels and non-participants at the 1%, 5% and 10% significance levels.
