# Supplementary material for: The impact of ICT-enabled extension campaign on farmers’ knowledge and management of fall armyworm in Uganda
Source: PLoS One. 2019 Aug 21;14(8):e0220844. doi: 10.1371/journal.pone.0220844 (PMC6703685; doi:10.1371/journal.pone.0220844)
Supplement: S2 Table — (DOCX) [file pone.0220844.s004.docx]

S2 Table.

|  | Radio only | Video only | Radio+  Video | Radio+  SMS | Radio+SMS  +Video |
| --- | --- | --- | --- | --- | --- |
| Age | 0.007 | -0.001 | -0.008 | -0.063** | -0.017 |
|  | (0.009) | (0.013) | (0.013) | (0.029) | (0.022) |
| Gender | -0.145 | 0.602 | 0.305 | -0.536 | 0.914 |
|  | (0.327) | (0.495) | (0.467) | (0.760) | (1.107) |
| Education | 0.005 | -0.017 | -0.040 | -0.058 | -0.062 |
|  | (0.035) | (0.049) | (0.045) | (0.082) | (0.078) |
| Household size | -0.038 | -0.060 | 0.035 | 0.101 | -0.053 |
|  | (0.039) | (0.055) | (0.046) | (0.092) | (0.101) |
| Dependency ratio | -0.201** | -0.123 | -0.274** | -0.212 | -0.279 |
|  | (0.102) | (0.138) | (0.138) | (0.256) | (0.309) |
| Land holding | -0.014 | 0.003 | 0.012 | -0.019 | 0.039 |
|  | (0.023) | (0.037) | (0.023) | (0.060) | (0.024) |
| Input market | 0.048** | -0.029 | 0.022 | 0.036 | -0.018 |
|  | (0.023) | (0.044) | (0.031) | (0.055) | (0.069) |
| Radio | 2.020*** | 0.315 | 2.591*** | 2.095* | 1.681 |
|  | (0.370) | (0.418) | (0.658) | (1.106) | (1.130) |
| Phone | 0.619 | 1.229** | 0.700 | 1.341 | 13.287 |
|  | (0.393) | (0.568) | (0.548) | (1.149) | (460.132) |
| Extension access | 0.319 | 1.127*** | 0.994*** | 1.101** | 1.655*** |
|  | (0.303) | (0.367) | (0.346) | (0.547) | (0.552) |
| Farmer group | 0.342 | 0.958** | 0.838** | 1.365*** | -0.205 |
|  | (0.295) | (0.388) | (0.343) | (0.529) | (0.636) |
| Off-farm activity | 0.193 | 0.372 | 0.139 | -0.415 | -0.265 |
|  | (0.246) | (0.337) | (0.306) | (0.517) | (0.533) |
| PPI | -0.010 | -0.049*** | -0.023 | -0.004 | 0.004 |
|  | (0.013) | (0.019) | (0.017) | (0.028) | (0.029) |
| Risk preference | 0.060 | 0.032 | 0.105** | 0.051 | 0.020 |
|  | (0.041) | (0.059) | (0.052) | (0.090) | (0.089) |
| District | 1.023*** | -0.683* | 0.812** | 1.895*** | 1.514** |
|  | (0.258) | (0.352) | (0.329) | (0.708) | (0.697) |
| Constant | -2.247*** | -0.213 | -3.470*** | -3.957* | -17.326 |
|  | (0.755) | (1.020) | (1.078) | (2.044) | (460.136) |

Note: ***, **, * denote 1%, 5%, and 10% significance level, respectively.
