# Supplementary material for: The impact of ICT-enabled extension campaign on farmers’ knowledge and management of fall armyworm in Uganda
Source: PLoS One. 2019 Aug 21;14(8):e0220844. doi: 10.1371/journal.pone.0220844 (PMC6703685; doi:10.1371/journal.pone.0220844)
Supplement: S3 Table — (DOCX) [file pone.0220844.s005.docx]

S3 Table.

|  | Before matching | | |  | After matching | | |
| --- | --- | --- | --- | --- | --- | --- | --- |
|  | Pseudo R^2^ | Mean bias | LR *χ*^2^  P-value |  | Pseudo R^2^ | Mean bias | LR *χ*^2^  P-value |
| Participants vs. non-participants | 0.133 | 25.9 | 0.000 |  | 0.012 | 4.9 | 0.541 |
| Radio only vs. non-participants | 0.168 | 26.9 | 0.000 |  | 0.019 | 5.4 | 0.699 |
| Video only vs. non-participants | 0.179 | 19.7 | 0.000 |  | 0.019 | 5.8 | 1.000 |
| Radio+Video vs. non-participants | 0.302 | 32.0 | 0.000 |  | 0.008 | 4.2 | 1.000 |
| Radio+SMS vs. non-participants | 0.351 | 37.2 | 0.000 |  | 0.037 | 6.4 | 1.000 |
| Radio+SMS+Video vs. non-participants | 0.334 | 35.5 | 0.003 |  | 0.065 | 8.8 | 1.000 |
| Video only vs. Radio only | 0.302 | 32.0 | 0.000 |  | 0.008 | 4.2 | 1.000 |
| Radio+Video vs. Radio only | 0.088 | 16.2 | 0.005 |  | 0.003 | 2.2 | 1.000 |
| Radio+SMS vs. Radio only | 0.126 | 18.2 | 0.187 |  | 0.011 | 4.7 | 1.000 |
| Radio+SMS+Video vs. Radio only | 0.206 | 22.9 | 0.022 |  | 0.004 | 3.1 | 1.000 |
| Passive Radio vs. non-participants | 0.177 | 26.0 | 0.009 |  | 0.009 | 4.3 | 0.998 |
| Active Radio vs. non-participants | 0.215 | 30.3 | 0.000 |  | 0.014 | 5.8 | 1.000 |
| Active Radio vs. Passive Radio | 0.108 | 18.9 | 0.011 |  | 0.006 | 3.0 | 1.000 |
