# Supplementary material for: The impact of ICT-enabled extension campaign on farmers’ knowledge and management of fall armyworm in Uganda
Source: PLoS One. 2019 Aug 21;14(8):e0220844. doi: 10.1371/journal.pone.0220844 (PMC6703685; doi:10.1371/journal.pone.0220844)
Supplement: S4 Table — (DOCX) [file pone.0220844.s006.docx]

S4 Table.

|  | Kernel matching | | |  | Doubly robust estimator | | |
| --- | --- | --- | --- | --- | --- | --- | --- |
|  | ATT | SE | ATT in % |  | ATT | SE | ATT in % |
| Regular monitoring | 0.23*** | 0.05 | 32.86 |  | 0.22*** | 0.05 | 30.99 |
| Use of chemical pesticides | 0.09 | 0.06 | 14.06 |  | 0.10* | 0.05 | 15.15 |
| Early planting | 0.23*** | 0.06 | 58.97 |  | 0.22*** | 0.06 | 56.41 |
| Handpicking of larvae | 0.22*** | 0.06 | 66.67 |  | 0.20*** | 0.06 | 60.61 |
| Frequent weeding | 0.21*** | 0.05 | 123.53 |  | 0.21*** | 0.04 | 131.25 |
| Destroying of infected plants | 0.14** | 0.05 | 60.87 |  | 0.15*** | 0.05 | 71.43 |
| Rotate with non-host crop | 0.13*** | 0.04 | 86.67 |  | 0.14*** | 0.04 | 100.00 |
| Use of local innovations | 0.07 | 0.05 | 36.84 |  | 0.03 | 0.04 | 15.00 |
| Fertilization | 0.11*** | 0.03 | 122.22 |  | 0.11*** | 0.04 | 110.00 |
| Intercropping | 0.08** | 0.03 | 160.00 |  | 0.06** | 0.03 | 100.00 |

Note: ***, **, * denote 1%, 5%, and 10% significance level, respectively.
