# Supplementary material for: The impact of ICT-enabled extension campaign on farmers’ knowledge and management of fall armyworm in Uganda
Source: PLoS One. 2019 Aug 21;14(8):e0220844. doi: 10.1371/journal.pone.0220844 (PMC6703685; doi:10.1371/journal.pone.0220844)
Supplement: S5 Table — (DOCX) [file pone.0220844.s007.docx]

S5 Table.

|  | Nearest neighbour matching | | | |  | Radius matching | | | |
| --- | --- | --- | --- | --- | --- | --- | --- | --- | --- |
|  | ATT | SE | ATT in % | Γ |  | ATT | SE | ATT in % | Γ |
| ***Participants vs. non-participants*** | |  |  |  |  |  |  |  |  |
| FAW identification score | 1.46*** | 0.35 | 24.74 | 5.40ꟷ5.50 |  | 1.27*** | 0.25 | 20.22 | 5.00ꟷ5.10 |
| FAW monitoring score | 0.60*** | 0.21 | 16.62 | 3.50ꟷ3.60 |  | 0.50*** | 0.16 | 13.55 | 3.50ꟷ3.60 |
| FAW management score | 1.84*** | 0.42 | 21.00 | 3.50ꟷ3.60 |  | 1.49*** | 0.33 | 16.39 | 4.80ꟷ4.90 |
| Overall FAW knowledge score | 3.89*** | 0.81 | 21.00 | 6.10ꟷ6.20 |  | 3.26*** | 0.60 | 17.10 | 6.80ꟷ6.90 |
| Adoption of FAW mgt. practices | 1.67*** | 0.30 | 55.85 | 3.60ꟷ3.70 |  | 1.62*** | 0.24 | 52.77 | 5.20ꟷ5.30 |
| ***Radio vs. non-participants*** |  |  |  |  |  |  |  |  |  |
| FAW identification score | 1.02*** | 0.29 | 16.22 | 2.80ꟷ2.90 |  | 1.11*** | 0.28 | 17.90 | 3.40ꟷ3.50 |
| FAW monitoring score | 0.42** | 1.83 | 11.44 | 2.10ꟷ2.20 |  | 0.38** | 0.18 | 10.24 | 2.30ꟷ2.40 |
| FAW management score | 1.27*** | 0.38 | 14.19 | 2.80ꟷ2.90 |  | 1.23*** | 0.37 | 13.67 | 3.10ꟷ3.20 |
| Overall FAW knowledge score | 2.71*** | 0.69 | 14.32 | 3.60ꟷ3.70 |  | 2.72*** | 0.66 | 14.38 | 3.90ꟷ4.00 |
| Adoption of FAW mgt. practices | 1.35*** | 0.28 | 43.08 | 3.80ꟷ3.90 |  | 1.40*** | 0.27 | 46.20 | 4.20ꟷ4.30 |
| ***Video vs. non-participants*** |  |  |  |  |  |  |  |  |  |
| FAW identification score | 1.16*** | 0.39 | 17.71 | 2.90ꟷ3.00 |  | 1.34*** | 0.39 | 20.70 | 3.30ꟷ3.40 |
| FAW monitoring score | 0.31 | 0.25 | 8.33 | — |  | 0.23 | 0.25 | 6.10 | — |
| FAW management score | 1.75*** | 0.54 | 19.60 | 2.90ꟷ3.00 |  | 1.59*** | 0.53 | 17.73 | 2.80ꟷ2.90 |
| Overall FAW knowledge score | 3.22*** | 0.97 | 16.76 | 3.10ꟷ3.20 |  | 3.16*** | 0.95 | 16.44 | 3.40ꟷ3.50 |
| Adoption of FAW mgt. practices | 1.61*** | 0.41 | 57.71 | 2.40ꟷ2.50 |  | 1.11*** | 0.39 | 34.37 | 1.90ꟷ2.00 |
| ***Radio+Video vs. non-participants*** | |  |  |  |  |  |  |  |  |
| FAW identification score | 1.88*** | 0.45 | 29.56 | 4.50ꟷ4.60 |  | 1.90*** | 0.35 | 29.92 | 8.20ꟷ8.30 |
| FAW monitoring score | 0.90*** | 0.28 | 24.73 | 3.10ꟷ3.20 |  | 0.92*** | 0.21 | 25.48 | 11.6ꟷ11.7 |
| FAW management score | 2.88*** | 0.64 | 33.22 | 4.60ꟷ4.70 |  | 2.47*** | 0.49 | 27.20 | 7.60ꟷ7.70 |
| Overall FAW knowledge score | 5.67*** | 1.18 | 30.37 | 7.10ꟷ7.20 |  | 5.29*** | 0.86 | 27.78 | 15.1ꟷ15.2 |
| Adoption of FAW mgt. practices | 1.81*** | 0.47 | 54.52 | 2.30ꟷ2.40 |  | 2.00*** | 0.38 | 63.90 | 4.40ꟷ4.50 |
| ***Radio+SMS vs. non-participants*** | |  |  |  |  |  |  |  |  |
| FAW identification score | 1.82*** | 0.50 | 29.17 | 5.50ꟷ5.60 |  | 2.26*** | 0.56 | 38.97 | 5.40ꟷ5.50 |
| FAW monitoring score | 0.91*** | 0.30 | 25.92 | 2.30ꟷ2.40 |  | 1.00*** | 0.32 | 29.33 | 3.20ꟷ3.30 |
| FAW management score | 1.89*** | 0.65 | 21.60 | 2.90ꟷ3.00 |  | 2.05*** | 0.70 | 23.84 | 2.40ꟷ2.50 |
| Overall FAW knowledge score | 4.62*** | 0.99 | 24.99 | 7.90ꟷ8.00 |  | 5.31*** | 1.17 | 29.81 | 7.90ꟷ8.00 |
| Adoption of FAW mgt. practices | 1.92*** | 0.67 | 58.90 | 2.40ꟷ2.50 |  | 1.85*** | 0.69 | 55.56 | 2.00ꟷ2.10 |
| ***Radio+SMS+Video vs. non-participants*** | | |  |  |  |  |  |  |  |
| FAW identification score | 2.02*** | 0.53 | 33.33 | 6.10ꟷ6.20 |  | 2.10*** | 0.53 | 35.12 | 3.80ꟷ3.90 |
| FAW monitoring score | 1.02*** | 0.27 | 28.98 | 2.80ꟷ2.90 |  | 1.06*** | 0.25 | 30.46 | 3.40ꟷ3.50 |
| FAW management score | 2.52*** | 0.69 | 30.00 | 3.40ꟷ3.50 |  | 2.55*** | 0.70 | 30.43 | 3.60ꟷ3.70 |
| Overall FAW knowledge score | 5.55*** | 1.08 | 30.87 | 5.30ꟷ5.40 |  | 5.71*** | 1.06 | 32.02 | 5.30ꟷ5.40 |
| Adoption of FAW mgt. practices | 3.02*** | 0.66 | 101.34 | 5.30ꟷ5.40 |  | 2.87*** | 0.67 | 91.37 | 6.10ꟷ6.20 |

Notes: *** and ** denote 1% and 5% significance level, respectively. Adoption of FAW mgt. practices = the number of FAW management practices adopted by a household. Γ= Critical level of hidden bias.
