# Supplementary figures and images for: The impact of ICT-enabled extension campaign on farmers’ knowledge and management of fall armyworm in Uganda
Source: PLoS One. 2019 Aug 21;14(8):e0220844. doi: 10.1371/journal.pone.0220844 (PMC6703685; doi:10.1371/journal.pone.0220844)

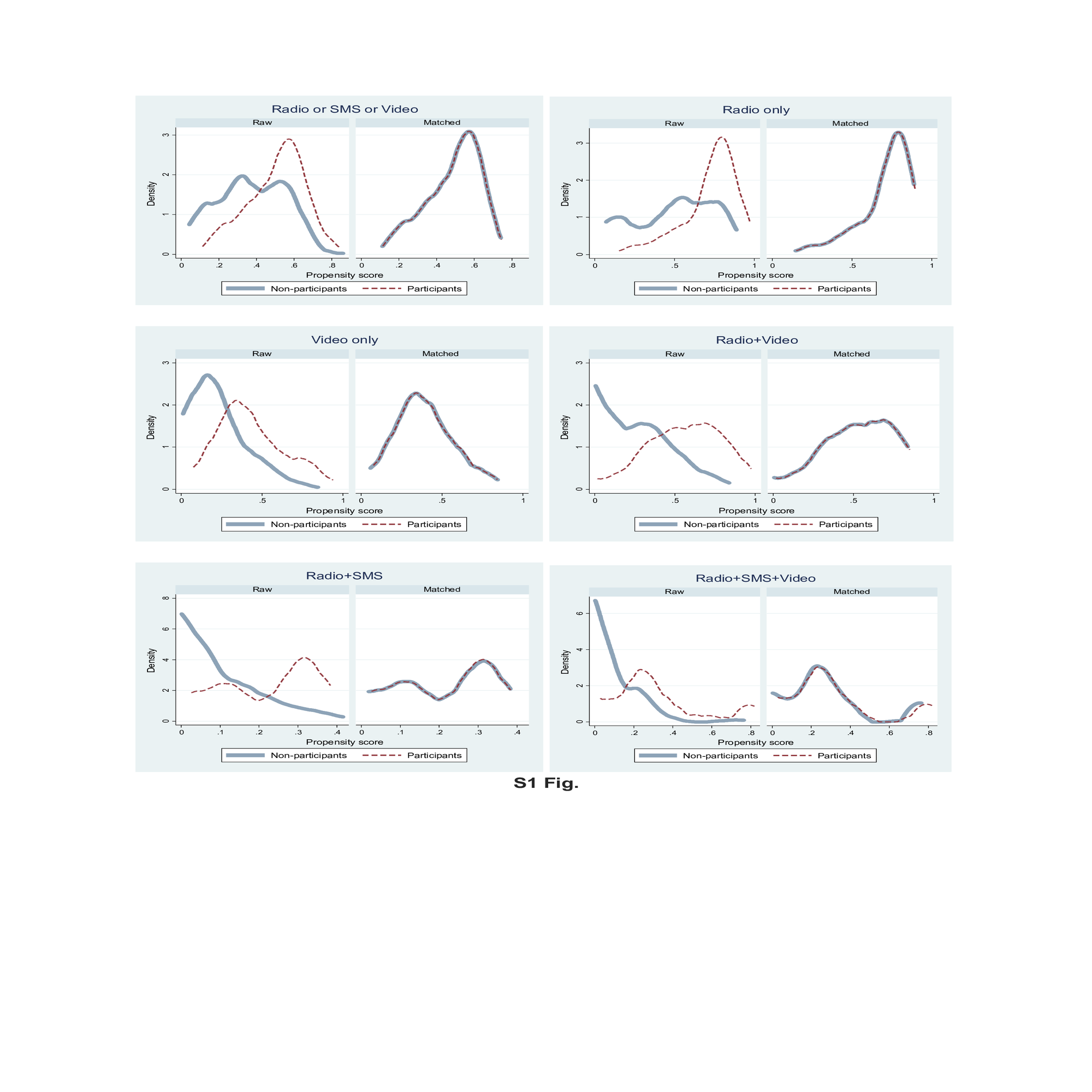

Supplement: S1 Fig — (TIFF) [file pone.0220844.s008.tiff]
